# Supplementary figures and images for: Structural characterization of ligand binding and pH-specific enzymatic activity of mouse Acidic Mammalian Chitinase
Source: eLife. 2024 Jun 17;12:RP89918. doi: 10.7554/eLife.89918 (PMC11182645; doi:10.7554/eLife.89918)

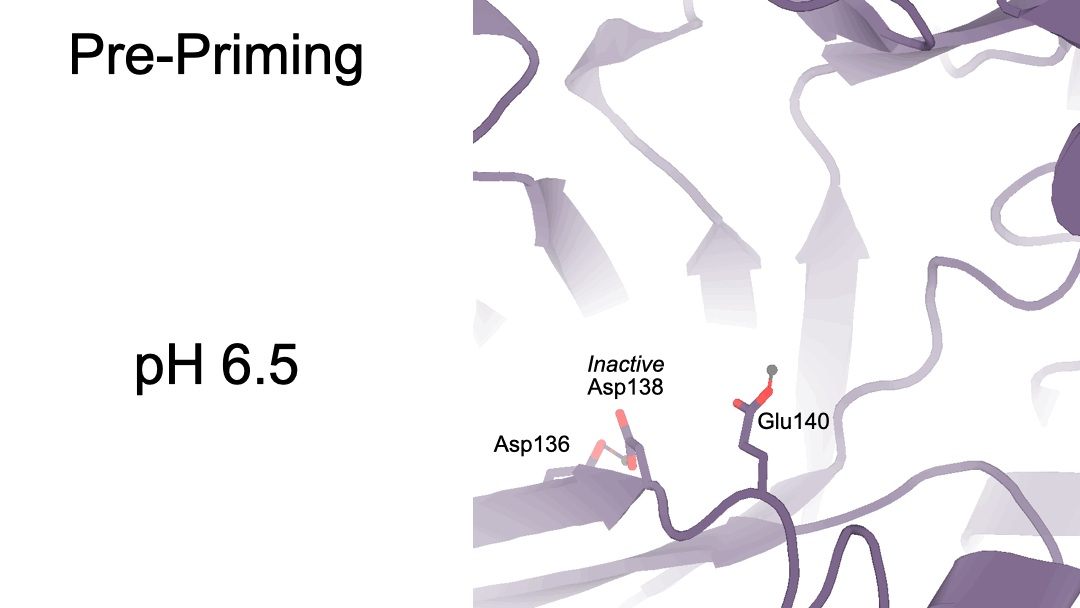

Supplement: Supplementary file 1 [file elife-89918-animation1.gif]

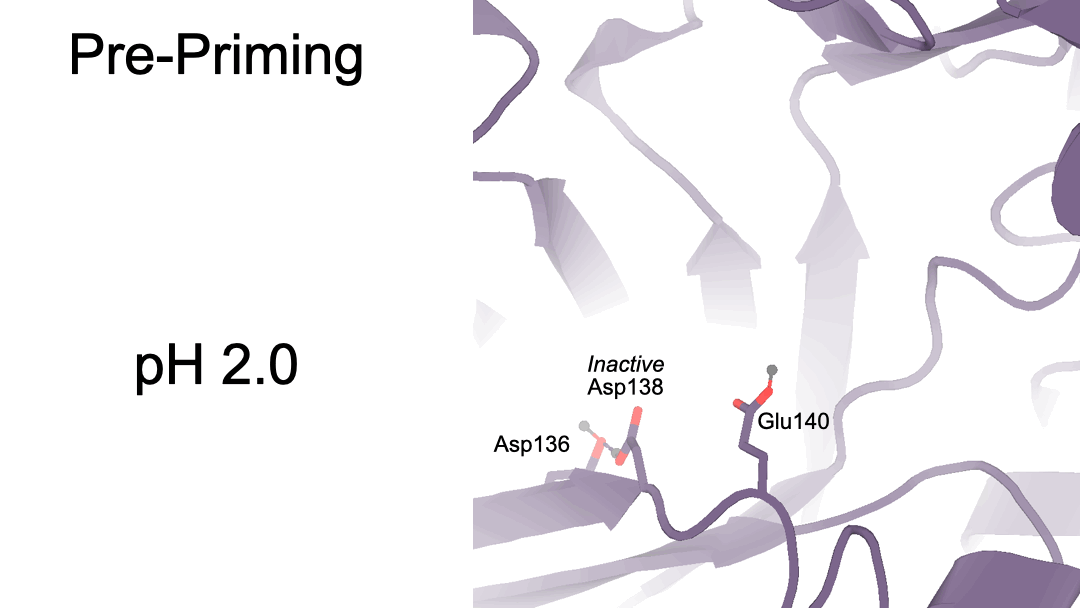

Supplement: Supplementary file 2 [file elife-89918-animation2.gif]
